# Amputation of the Thigh in Civil and Military Practice

**Published:** 1855-05

**Authors:** Richard McSherry

**Affiliations:** Baltimore


### RECORD OF MEDICAL SCIENCE.

As our limits do not permit us to copy as many of the papers published in other Journals as we could wish, we shall occasionally take the opportunity of laying a short abstract of such of them as we deem most important, before our readers. We shall confine ourselves, in this number, entirely to surgical notices.

*Amputation of the Thigh in Civil and Military Practice. The comparative success of Secondary Amputation.* By RICHARD McSHERRY, M. D. of Baltimore. (Amer. Jour. of Med. Sciences, January, 1855.)—The great fatality which attends upon amputation of the thigh after gun shot wounds is evidenced by the fact, that, during the campaign in Mexico, under Gen. Scott, there were no recoveries, so far as Dr. McSherry could learn, after the operation. This mortality is not peculiar to our country, as Mr. Ribes examined four thousand invalid soldiers, among all of whom he did not find one single instance of injury of the femur by shot, nor one who had undergone amputation of the thigh. Malgaigne was equally unfortunate with all his cases during the Polish campaign. In civil life, the case is widely different. The statistical tables, published by Dr. Norris, state that during a period of ten years, of sixteen amputations of the thigh, performed in the Pennsylvania Hospital, fourteen recovered, and but two died. The two fatal cases were primary amputations, after fracture of the thigh and compound and comminuted fracture of the leg, respectively. Of the fourteen successful cases, seven were fractures (one ununited fracture of thigh), one tumor on the knee, and six caries of knee-joint. These results are favorable, Dr. McS. thinks, to secondary amputations.

In the Parisian hospitals, there were, in ten years, forty-four amputations of the thigh. Of these, thirty-four died, and ten recovered. All of these, however, were amputations after *traumatic* lesions. A comparison of these results exhibits, Dr. McSherry observes, that patients who undergo great operations, in full robust health, are more liable to perish than those who are subjected to them for chronic disease. There is abundant proof also, that delay *is* advantageous. Dupuytren, in 1830, treated thirteen cases of fracture of the thigh *without* amputation; of these six recovered, and eight died. Malgaigne reports five cases treated by himself, *without* amputation, of whom two recovered, and three died. These results, compared with those in the same hospitals after amputation, (see ante,) show that the chances of life were greatest where the operation was not only deferred, but forgone altogether. Dr. McSherry considers that these facts show that the rule that the thigh must be amputated immediately upon fracture of the femur by shot, is a bad one, inasmuch as it requires certain destruction of the limb, without adding appreciably to the chances of ultimate recovery. When there is thorough disorganization of the limb from injury, the case, of course, is different. The *exception*, however, should not be made the *rule*.

